# Spontaneous lung colonization in the cystic fibrosis rat model is linked to gastrointestinal obstruction

**DOI:** 10.1128/mbio.03883-24

**Published:** 2025-03-05

**Authors:** Mikayla Murphree-Terry, Johnathan D. Keith, Ashley M. Oden, Susan E. Birket

**Affiliations:** 1 Department of Medicine, Division of Pulmonary, Allergy, and Critical Care Medicine, University of Alabama at Birmingham, Birmingham, Alabama, USA; 2 Gregory Fleming James Cystic Fibrosis Research Center, University of Alabama at Birmingham, Birmingham, Alabama, USA; The University of Mississippi Medical Center, Jackson, Mississippi, USA

**Keywords:** microbiome, airway colonization, cystic fibrosis, mucus, DIOS

## Abstract

Cystic fibrosis (CF) is a genetic disease caused by mutations in the cystic fibrosis transmembrane conductance regulator (*CFTR*) gene, resulting in CFTR protein dysfunction. CFTR dysfunction has multi-organ consequences, leading to dehydrated mucus that is adherent to epithelia. In the lungs, this leads to recalcitrant infections with bacteria such as *Pseudomonas aeruginosa*. In the gut, mucus-laden feces can adhere to the intestines, resulting in distal intestinal obstruction syndrome (DIOS). There is limited information on how lung colonization and DIOS are correlated in people with CF (pwCF). In this novel work, we describe the development of spontaneous lung colonization of CF pathogens in young (<3 months old) CF rats, preceding the development of DIOS. Once DIOS is established, the lung microbiome becomes predominated by taxa also observed in the feces. Induced infection with *P. aeruginosa* in the CF rats reflects data found in pwCF, as once CF rats are infected, they retain a higher relative abundance of *P. aeruginosa* than their healthy agemates. Finally, we found that ivacaftor treatment favors a healthier gut microbiome in CF rats, decreasing the relative abundance of *Escherichia coli*. These results indicate that the CF rat model is recapitulative of human CF disease with the spontaneous lung colonization of traditional CF pathogens and maintenance of *P. aeruginosa* after induced infection. Furthermore, these results indicate a possible role for the gut-lung axis in lung colonization and DIOS in CF.

**IMPORTANCE:**

These data describe for the first time the development of spontaneous lung colonization in the cystic fibrosis (CF) rat model, a hallmark aspect of human CF disease. We also find that CF rats infected with *Pseudomonas aeruginosa* maintain higher relative abundance following chronic infection as compared to healthy rats, similar to those is seen in people with CF. Additionally, we describe the possible contribution of the gut-lung axis linking lung health with distal intestinal obstruction syndrome, a relationship largely unexplored in the context of CF.

## INTRODUCTION

Cystic fibrosis (CF) is an autosomal recessive genetic disease caused by mutations in the cystic fibrosis transmembrane conductance regulator (*CFTR*) gene. Mutations in this gene result in an absent or dysfunctional CFTR protein (1). Loss of CFTR function contributes to the accumulation of viscous mucus that becomes adherent to the epithelium in multiple organ systems, including the respiratory and digestive tracts (2). This stationary mucus predisposes people with cystic fibrosis (pwCF) to developing recalcitrant bacterial infections.

Lung function and pathogen infection are two major determinants of morbidity and mortality among pwCF (3). PwCF in their first decade of life exhibit moderate diversity of microbial taxa in the lung; colonization from genera such as *Streptococcus*, *Prevotella*, *Rothia*, *Veillonella*, and *Haemophilus* is commonly observed (4, 5). *Streptococcus* predominates, with *Staphylococcus* sp. and *Pseudomonas aeruginosa* comprising about half of the total lung microbiome (6). As pwCF age, their lung microbiome becomes less diverse as the traditional CF pathogens, *P. aeruginosa* and *S. aureus*, become more abundant, with *Stenotrophomonas maltophilia*, *Achromobacter* sp., and *Burkholderia cepacia* complex colonizing to a lesser extent (7). This decrease in microbial diversity is associated with declining lung function (8, 9).

An additional complication of CF is the development of distal intestinal obstruction syndrome (DIOS), a condition where thick mucus mixes with feces and adheres to the intestinal wall. DIOS occurs in approximately 35.5 episodes per 1,000 patient-years in adults and can negatively affect nutritional status through appetite suppression and nutrient malabsorption (10). Like pwCF, the CF rat model experiences intestinal obstruction, with approximately 70% of these rats becoming obstructed after weaning. By diet supplementation with laxatives and soft food, CF rat survival is increased, and intestinal complications are reduced (11–13). As poor nutritional status is another key mediator of morbidity and mortality for pwCF (3), an animal model that accurately manifests the intestinal phenotype is beneficial in investigating how intestinal obstruction correlates with respiratory colonization.

Because CF is a multi-organ disease that impacts both the respiratory and digestive systems, it is imperative to consider how microbial and immune interactions between the two systems, termed the gut-lung axis, influence clinical outcomes. Interactions between the immune system and the microbiome are a two-way process in both the lung and gut. The lung and gut microbiomes both modulate their local immune systems, aiding in maturation and migration of immune cells; inversely, inflammation can drastically change the microbial composition of the two systems (14). The mucosal surfaces of the lung and gut are similar in composition and function. Immunological and metabolomic crosstalk between the two systems, as well as direct transmission of microbes, has been observed (15). Furthermore, it has been shown that gut dysbiosis in pwCF is associated with poor pulmonary outcomes, namely exacerbations (15, 16). Thus, it is important to consider how the gut-lung axis is involved in pulmonary and gastrointestinal (GI) manifestations of CF.

Highly effective modulator therapies, including ivacaftor, restore CFTR function, and improve pulmonary function (17). Ivacaftor also improves GI function. Ivacaftor treatment increases GI pH and body weight, decreases intestinal inflammation, and improves nutritional status in pwCF, but symptoms such as pain and regional GI transit times are not altered (17–20). Furthermore, ivacaftor has not been shown to impact the development of constipation or DIOS in pwCF. While ivacaftor administration to our humanized-G551D CFTR rat model of CF has been shown to increase airway surface liquid (ASL) depth, increase mucociliary transport (MCT) rate, decrease mucus viscosity (21), and partially resolve inflammation (22), the effect of ivacaftor on the lung and gut microbiomes has not been studied in this model.

Here, we describe for the first time the identification of changes to the lung microbiome of CF rats, both before the development of DIOS, after the development of DIOS, and following ivacaftor therapy. We hypothesized that, as in pwCF, there will be age-related differences in the microbiome of CF rat lungs. In this study, we aimed to assess if the development of DIOS in CF rats correlated with the presence of lung bacterial colonization.

## RESULTS

### The lung microbiome is not dependent on CFTR genotype

**Fig 1:**
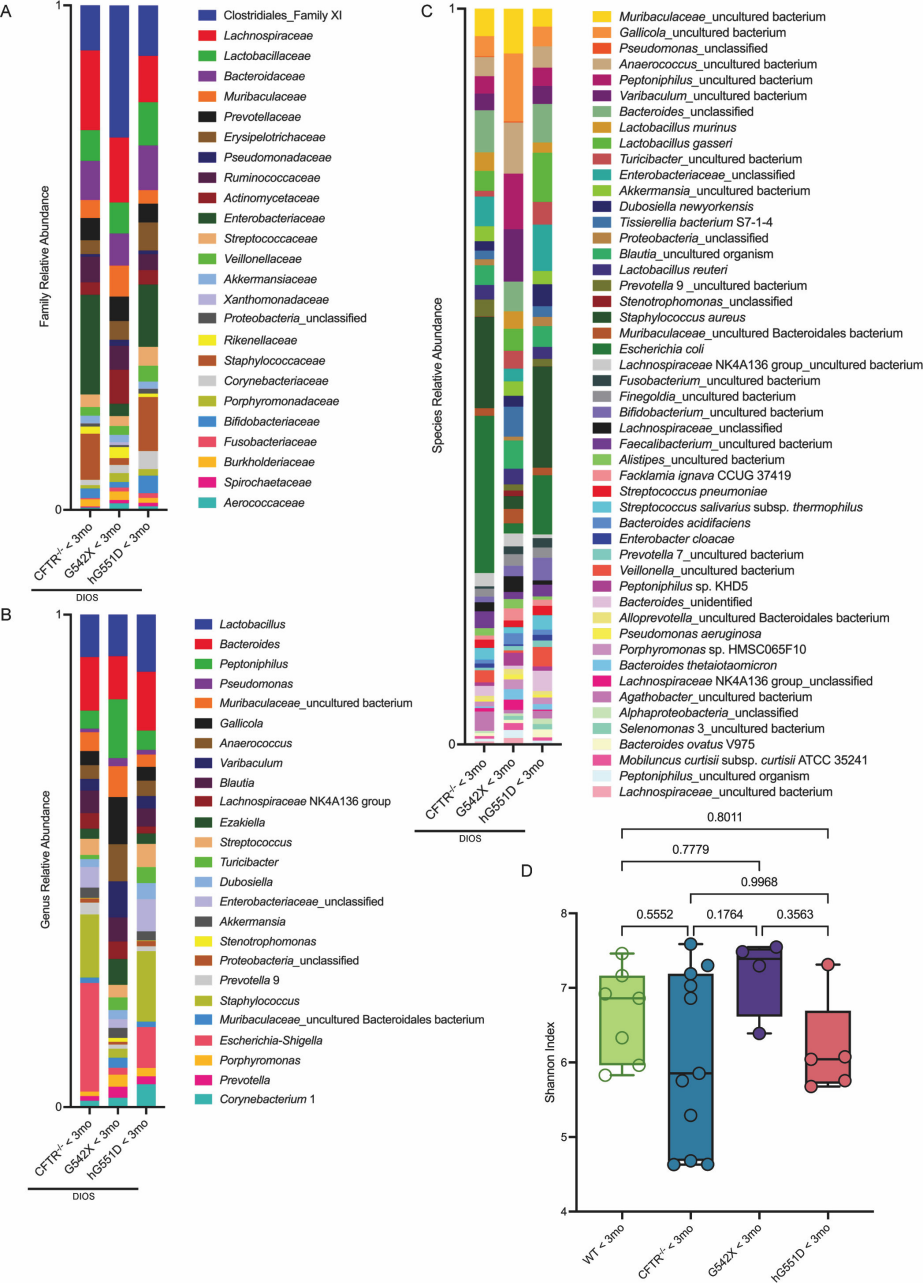
CFTR genotype does not significantly alter relative abundance of lung bacteria taxa. Relative family (**A**) and genus (**B**) level taxonomic abundance as determined by amplicon sequence variants (ASVs) are shown. The top 25 ASVs excluding mitochondria are included. Relative species (**C**) level taxonomic abundance as determined by ASVs is shown. The top 50 ASVs excluding mitochondria are included. Alpha diversity (**D**) measured by the Shannon index is reported. Data are shown as maximum, median, minimum, and interquartile range (IQR). Data are not significant (*P* values listed), determined by one-way ANOVA with Tukey’s multiple comparison test. CFTR^−/−^ < 3 mo *n* = 11, G542X < 3 mo *n* = 4, hG551D < 3 mo *n* = 5, WT *n* = 5.

First, we asked if the lung microbiome is dependent on CFTR mutation, as has been suggested to be the case in human studies (6). As demonstrated previously, rats with the CFTR Δ16 bp mutation, the humanized-G551D CFTR insertion, and the G542X CFTR point mutation all have similar lung and GI phenotypes, compared to the wild type (WT) (11–13). We also showed previously that at 3 months of age, mucus begins to accumulate in the lungs (21). To identify if there were any notable findings in the lung microbiome prior to progression of lung disease, we assayed CF rats younger than 3 months old (<3 mo). We found that the composition of the lung microbiome was similar among the KO, hG551D, and G542X genotypes at the family (Fig. 1A), genus (Fig. 1B), and species (Fig. 1C) taxonomic levels. Additionally, we observed no difference in microbial diversity by Shannon index among the genotypes (Fig. 1D), or compared to WT. These results revealed that the lung microbiome is not dependent on CFTR mutation in the rat models. Therefore, we have grouped all genotypes in subsequent figures unless otherwise stated.

### Paired CF rat lung and feces exhibit similar microbial communities after the development of DIOS

**Fig 2:**
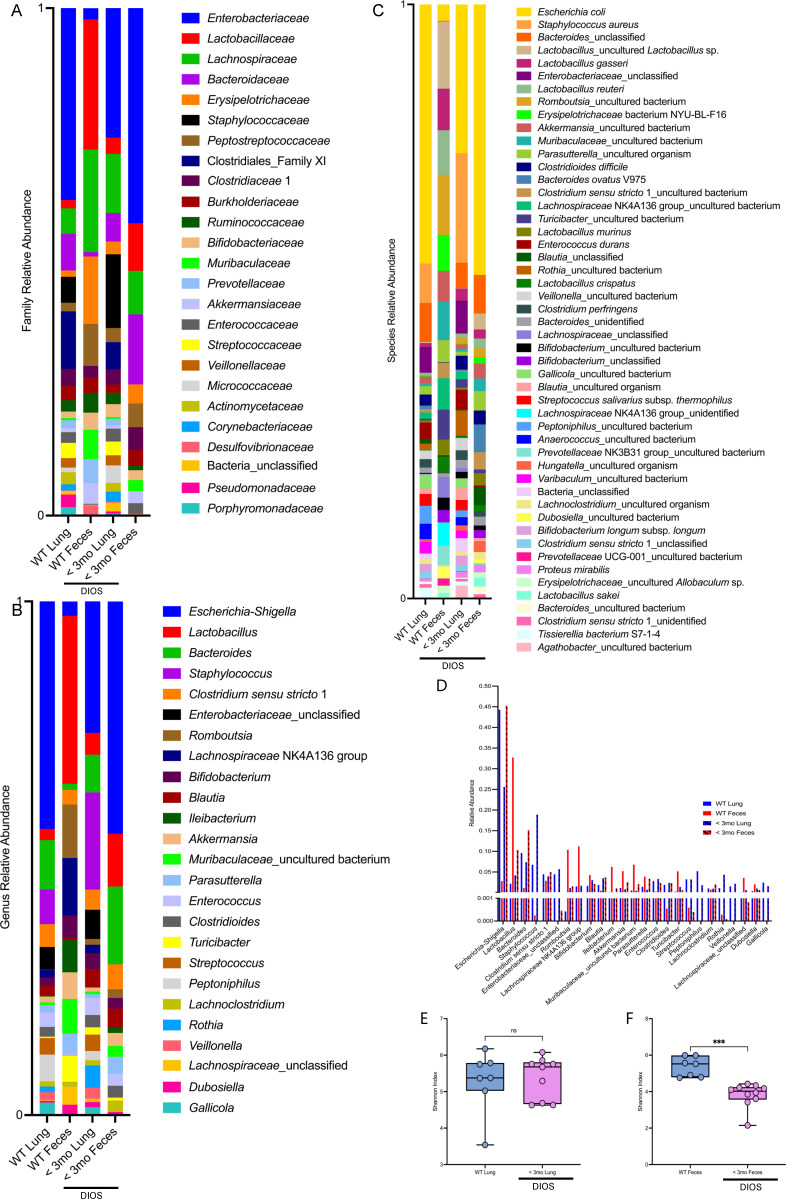
Microbiome analysis of paired rat lung tissue and feces reveals similar microbial communities and fecal dysbiosis in the CF rats after the development of distal intestinal obstruction syndrome (DIOS). Relative family (**A**) and genus (**B**) level taxonomic abundance as determined by ASVs are shown. The top 25 ASVs excluding mitochondria and chloroplast are included. Relative species (**C**) level taxonomic abundance as determined by ASVs is shown. The top 50 ASVs excluding mitochondria and chloroplast are included. Relative abundance of lung and fecal taxa is displayed for both WT and <3 mo CF rats (**D**). Alpha diversity of lung tissue (**E**) measured by the Shannon index is reported. Data are shown as maximum, median, minimum, and IQR. Data are ns, determined by unpaired *t* test. Alpha diversity of feces (**F**) as measured by the Shannon index is reported. Data are shown as maximum, median, minimum, and IQR and analyzed via Mann-Whitney test. ***P < 0.001. WT *n* = 7, <3 mo *n* = 9–10.

Although diet supplementation of CF rats reduces the morbidity and mortality from GI obstruction, it does not completely prevent the development of DIOS (11). Once young (<3 mo) CF rats were identified as experiencing GI distress, and DIOS was confirmed upon sacrifice, lung tissue and feces were collected to assess the microbiome after the development of DIOS. Similarly to the lung microbiome, we found that the fecal microbiome was not dependent on CFTR genotype (Fig. S1). Lung tissue and feces were also collected from healthy WT rats, as the non-disease comparator. There was greater overlap in the relative abundances of many fecal-associated microbes in the paired <3 mo lung and feces compared to the paired WT lung and feces at the family (Fig. 2A), genus (Fig. 2B), and species (Fig. 2C) taxonomic levels. This is demonstrated in plots comparing the relative abundances of each bacterial genus in the WT lung (blue bars), WT feces (red bars), <3 mo lung (blue bars with hashes), and <3 mo feces (red bars with hashes). When the relative abundance of each bacterial genus in the feces is compared to the relative abundance of that genus in the lung (feces/lung ratio), the mean of <3 mo CF rats is 1.45 (±2.02), while the mean of WT rats is 15.23 (±50.31) (Table 1). A mean of 1 indicates the same relative abundance of bacteria in the feces and lung; therefore, more bacterial taxa had similar abundance in the lung and feces in the <3 mo CF rats. We found no difference in the microbial diversity of the lung tissue (Fig. 2E). However, we observed dysbiosis in the feces of <3 mo rats (Table 2). At the genera level, the relative abundance of *Escherichia-Shigella* (Fig. S2A) was higher, while the relative abundance of *Lactobacillus* (Fig. S2B) and *Bifidobacterium* (Fig. S2C) was lower in the <3 mo rats as compared to WT rats. Fecal microbiome dysbiosis was further supported by decreased microbial diversity in the <3 mo feces (Fig. 2F). Taken together, these data indicate that the development of DIOS in the <3 mo rat is associated with GI dysbiosis, and there is greater overlap in the relative abundances of many fecal-associated microbes in the lung and feces of the <3 mo rat.

**TABLE 1:** Genus level bacterial taxon relative abundance ratios (feces/lung) of WT and <3 mo CF rats^*a*^

Genus	WT feces/lung ratio	<3 mo feces/lung ratio
*Escherichia-Shigella*	0.063310801	1.765899631
*Lactobacillus*	15.01288116	2.4378217
*Bacteroides*	0.117241111	2.053201972
*Staphylococcus*	0.003627524	0
*Clostridium sensu stricto* 1	0.646367918	1.259702152
*Enterobacteriaceae_*unclassified	0	0.00808466
*Romboutsia*	247.8930905	1.356138436
*Lachnospiraceae* NK4A136 group	7.401745901	0
*Bifidobacterium*	2.605492368	0.688859841
*Blautia*	0.270879165	1.052471464
*Ileibacterium*	36.6180973	8.512481774
*Akkermansia*	4.52232074	3.164875942
*Muribaculaceae_*uncultured bacterium	13.19186601	4.37599397
*Parasutterella*	2.703250879	4.723328732
*Enterococcus*	0.089429769	0.691726952
*Clostridioides*	0.028996672	0.967665561
*Turicibacter*	19.62974997	0.434361617
*Streptococcus*	0.018042396	0.012221339
*Peptoniphilus*	0	0
*Lachnoclostridium*	0.740916214	2.060858477
*Rothia*	0.02554754	0.001969639
*Veillonella*	0.001565137	0
*Lachnospiraceae_*unclassified	23.72657739	0.127286161
*Dubosiella*	5.557676955	0.644398514
*Gallicola*	0	0
Mean	**15.23474694^*b*^**	**1.453573941**
Median	**0.693642066**	**0.829696256**
SD	**50.31061847**	**2.016051233**

^*a*^ The average relative abundance of each bacterial taxon was calculated for the feces and lung tissue of WT and <3 mo CF rats. The relative abundance of the bacterial taxon in the feces was compared to the relative abundance of the bacterial taxon in the lung tissue. Mean, median, and SD of the ratios were calculated for WT and <3 mo CF rats.

^*b*^ Bold values indicates ns (*P* = 0.5575).

**TABLE 2:** Percentages of the top 25 bacterial taxa in the WT and <3 mo lung and feces at the family taxonomic level^*a*^

Family	WT lung	WT feces	<3 mo lung	<3 mo feces
*Enterobacteriaceae*	37.8%	2.2%	25.5%	42.4%
*Lactobacillaceae*	1.7%	25.7%	3.2%	9.4%
*Lachnospiraceae*	5.0%	20.2%	11.7%	8.5%
*Bacteroidaceae*	7.2%	0.9%	5.6%	13.8%
*Erysipelotrichaceae*	1.2%	13.3%	2.5%	3.8%
*Staphylococcaceae*	5.1%	0.0%	14.5%	0.0%
*Peptostreptococcaceae*	1.7%	8.2%	2.8%	4.7%
Clostridiales_Family XI	11.3%	0.0%	5.4%	0.0%
*Clostridiaceae* 1	3.4%	2.3%	3.0%	4.5%
*Burkholderiaceae*	2.8%	3.1%	1.6%	3.1%
*Ruminococcaceae*	2.2%	3.9%	2.2%	0.9%
*Bifidobacteriaceae*	1.3%	3.4%	2.5%	1.9%
*Muribaculaceae*	0.4%	5.8%	0.4%	2.2%
*Prevotellaceae*	1.5%	4.7%	1.3%	0.0%
*Akkermansiaceae*	0.9%	4.1%	0.6%	2.3%
*Enterococcaceae*	2.1%	0.2%	2.5%	2.1%
*Streptococcaceae*	3.0%	0.0%	2.7%	0.0%
*Veillonellaceae*	1.9%	0.0%	2.0%	0.0%
*Micrococcaceae*	0.9%	0.0%	3.5%	0.0%
*Actinomycetaceae*	2.4%	0.0%	1.7%	0.0%
*Corynebacteriaceae*	1.2%	0.0%	2.0%	0.0%
*Desulfovibrionaceae*	0.1%	2.1%	0.2%	0.3%
Bacteria_unclassified	0.7%	0.0%	1.7%	0.0%
*Pseudomonadaceae*	2.5%	0.0%	0.4%	0.0%
*Porphyromonadaceae*	1.7%	0.0%	0.4%	0.0%

^*a*^ The average relative abundance of each bacterial taxon at the family taxonomic level is shown here as percentage values.

### The lung microbiome of moribund CF rats is predominated by traditional CF pathogens

**Fig 3:**
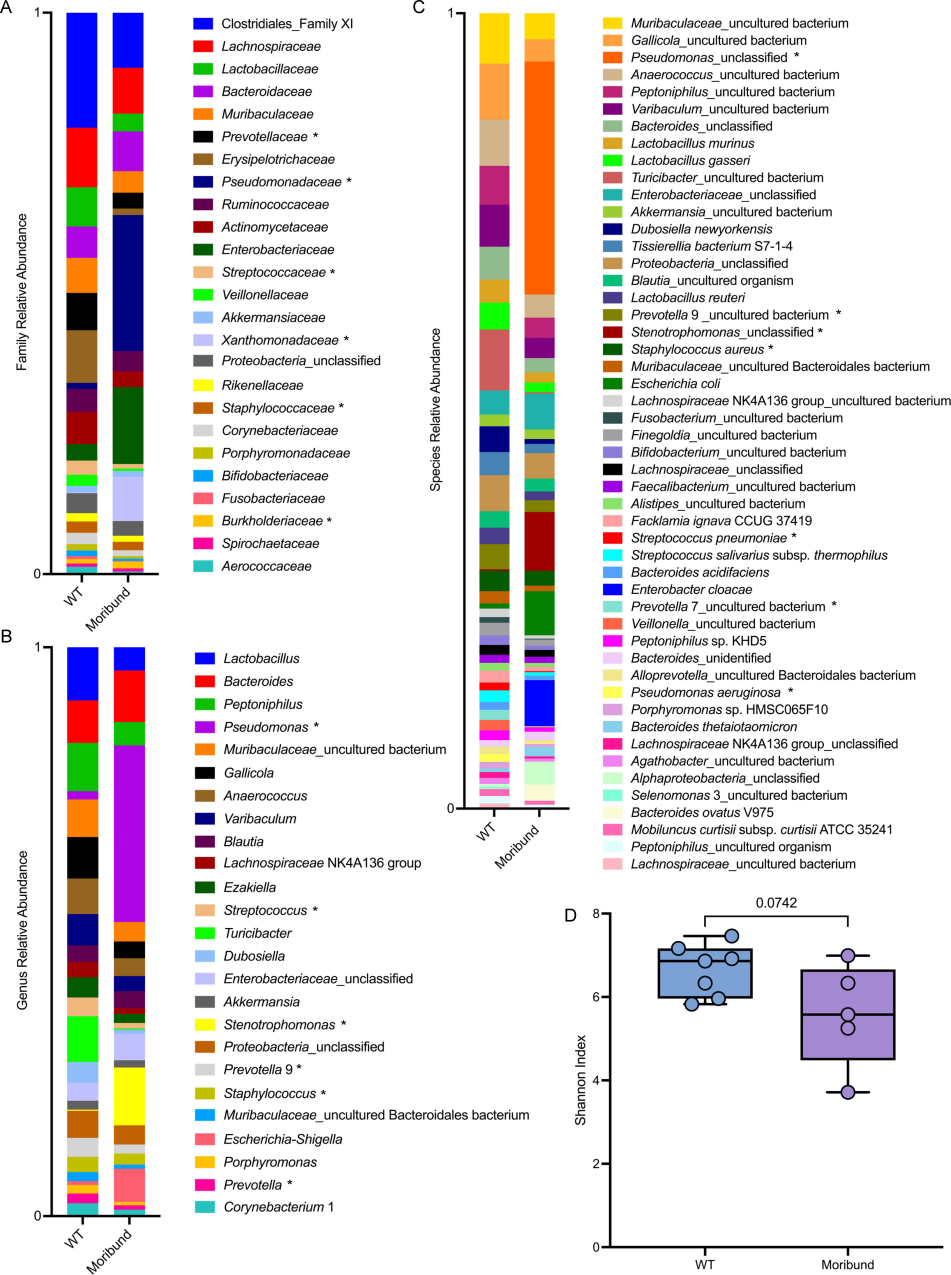
The CF rat lung microbiome is predominated by traditional CF pathogens prior to the development of DIOS. Relative family (**A**) and genus (**B**) level taxonomic abundance as determined by ASVs are shown. The top 25 ASVs excluding mitochondria are included. Relative species (**C**) level taxonomic abundance as determined by ASVs is shown. The top 50 ASVs excluding mitochondria are included. Alpha diversity (**D**) measured by the Shannon index is reported. Data are shown as maximum, median, minimum, and IQR. Data are ns, determined by unpaired *t* test. WT *n* = 7, Moribund *n* = 5. Traditional CF pathogens denoted with an asterisk.

Our lab noticed that 25% of the CF rats displayed signs of declining health; however, upon sacrifice, these rats had no evidence of DIOS or other GI abnormalities. We hypothesized that this disease stage, which we termed moribund, may induce changes to the lung microbiome. We found that the lung microbiome of moribund CF rats, compared to healthy WT rats, is predominated by traditional CF pathogens at the family (Fig. 3A), genus (Fig. 3B), and species (Fig. 3C) taxonomic levels. The families *Pseudomonadaceae*, *Xanthomonadaceae*, and *Burkholderiaceae* are more abundant in moribund CF rats (Fig. S2D), while *Prevotellaceae*, *Streptococcaceae*, and *Staphylococcaceae* are more abundant in WT rats (Fig. S2E). Moribund CF rats also exhibited a trending, though non-significant, decrease in microbial diversity of the lungs (Fig. 3D), consistent with what is observed with increasing age and lung disease severity in pwCF (23, 24). Percentage values of the top 25 bacterial taxa at the family (Table 3) and genus (Table 4) taxonomic levels are listed, confirming the predomination of traditional CF pathogens in moribund rats. These data are the first reported evidence of spontaneous colonization in the CF rat model and found that lung colonization with traditional CF pathogens occurs prior to the development of DIOS. Furthermore, certain pathogenic genera observed in the moribund CF rat lung, such as *Pseudomonas* and *Stenotrophomonas*, are the same as those found in the lungs of pwCF (6), further validating the CF rat as a model for human CF disease.

**TABLE 3:** Percentages of the top 25 bacterial taxa in the WT, moribund, <3 mo, and >3 mo rats at the family taxonomic level^*a*^

Family	WT	Moribund	<3 mo	>3 mo
Clostridiales_Family XI	20.5%	9.8%	12.7%	19.6%
*Lachnospiraceae*	10.6%	8.2%	13.6%	13.3%
*Lactobacillaceae*	7.0%	3.2%	6.7%	8.7%
*Bacteroidaceae*	5.6%	7.1%	7.7%	7.7%
*Muribaculaceae*	6.3%	3.8%	3.9%	7.0%
*Prevotellaceae*	6.6%	2.8%	4.3%	5.6%
*Erysipelotrichaceae*	9.4%	1.2%	3.7%	3.0%
*Pseudomonadaceae*	1.1%	24.2%	0.7%	1.2%
*Ruminococcaceae*	4.1%	3.6%	4.6%	5.1%
*Actinomycetaceae*	5.8%	2.8%	3.4%	5.5%
*Enterobacteriaceae*	2.9%	13.7%	14.4%	2.6%
*Streptococcaceae*	2.5%	0.8%	2.7%	1.6%
*Veillonellaceae*	2.0%	0.4%	2.0%	1.5%
*Akkermansiaceae*	1.2%	1.0%	1.5%	1.3%
*Xanthomonadaceae*	0.2%	7.9%	0.2%	3.4%
*Proteobacteria_*unclassified	3.6%	2.6%	0.6%	1.6%
*Rikenellaceae*	1.5%	1.1%	1.4%	2.6%
*Staphylococcaceae*	2.0%	1.5%	7.9%	1.6%
*Corynebacteriaceae*	2.0%	1.0%	1.8%	1.6%
*Porphyromonadaceae*	1.1%	0.5%	1.0%	1.2%
*Bifidobacteriaceae*	1.0%	0.4%	2.1%	1.5%
*Fusobacteriaceae*	0.5%	0.1%	0.5%	0.4%
*Burkholderiaceae*	0.8%	1.2%	1.4%	1.2%
*Spirochaetaceae*	0.6%	0.6%	0.4%	0.3%
*Aerococcaceae*	1.3%	0.5%	0.7%	1.0%

^*a*^ The average relative abundance of each bacterial taxon at the family taxonomic level is shown here as percentage values.

**TABLE 4:** Percentages of the top 25 bacterial taxa in the WT, moribund, <3 mo, and >3 mo rats at the genus taxonomic level^*a*^

Genus	WT	Moribund	<3 mo	>3 mo
*Lactobacillus*	9.3%	4.1%	9.3%	12.1%
*Bacteroides*	7.5%	9.1%	10.7%	10.7%
*Peptoniphilus*	8.5%	4.1%	5.4%	9.1%
*Pseudomonas*	1.5%	31.0%	1.0%	1.6%
*Muribaculaceae*_uncultured bacterium	6.6%	3.4%	4.0%	7.1%
*Gallicola*	7.3%	2.9%	4.2%	6.7%
*Anaerococcus*	6.3%	3.1%	3.9%	5.7%
*Varibaculum*	5.5%	2.7%	3.4%	6.1%
*Blautia*	2.9%	3.0%	4.4%	2.7%
*Lachnospiraceae* NK4A136 group	2.7%	1.0%	2.8%	5.3%
*Ezakiella*	3.5%	1.6%	2.7%	3.9%
*Streptococcus*	3.3%	0.9%	3.5%	2.1%
*Turicibacter*	8.0%	0.2%	1.8%	1.1%
*Dubosiella*	3.7%	0.7%	2.1%	2.1%
*Enterobacteriaceae_*unclassified	3.2%	4.7%	4.3%	2.1%
*Akkermansia*	1.5%	1.3%	2.0%	1.9%
*Stenotrophomonas*	0.2%	10.2%	0.2%	4.7%
*Proteobacteria_*unclassified	4.7%	3.4%	0.8%	2.3%
*Prevotella* 9	3.3%	1.6%	1.8%	1.6%
*Staphylococcus*	2.7%	1.9%	10.9%	2.3%
*Muribaculaceae_*uncultured Bacteroidales bacterium	1.6%	0.7%	1.3%	2.4%
*Escherichia-Shigella*	0.7%	5.8%	14.4%	1.3%
*Porphyromonas*	1.5%	0.6%	1.4%	1.7%
*Prevotella*	1.7%	0.8%	1.4%	1.7%
*Corynebacterium* 1	2.3%	1.1%	2.3%	1.7%

^*a*^ The average relative abundance of each bacterial taxon at the genus taxonomic level is shown here as percentage values.

### After DIOS, the CF lung microbiome is predominated by fecal microbes but still maintains age-based differences in traditional CF pathogens

**Fig 4:**
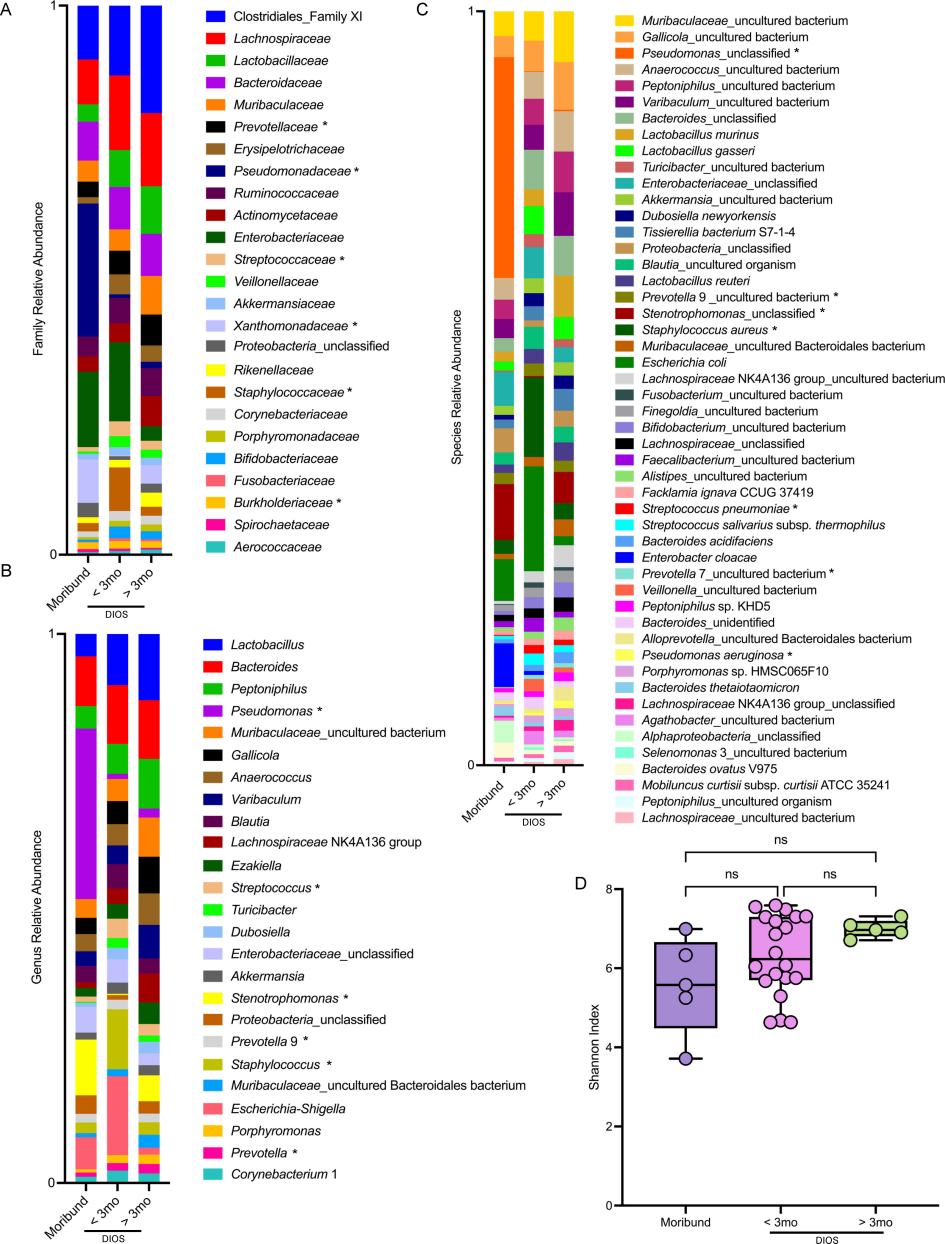
Key CF pathogens are still present in the lung after DIOS, with notable age-based differences in relative abundance. Relative family (**A**) and genus (**B**) level taxonomic abundance as determined by ASVs are shown. The top 25 ASVs excluding mitochondria are included. Relative species (**C**) level taxonomic abundance as determined by ASVs is shown. The top 50 ASVs excluding mitochondria are included. Alpha diversity (**D**) measured by the Shannon index is reported. Data are shown as maximum, median, minimum, and IQR. Data are ns, determined by Kruskal-Wallis test with Dunn’s multiple comparison test. Moribund *n* = 5, <3 mo *n* = 20, >3 mo *n* = 5. Traditional CF pathogens denoted with an asterisk.

After DIOS, the lung microbiome of both <3 mo and >3 mo (older than 3 months old) CF rats was predominated by fecal microbes at the family (Fig. 4A), genus (Family 4B), and species (Family 4C) taxonomic levels. *Lactobacillus*, *Bacteroides*, *Peptoniphilus*, and *Gallicola* were more abundant in >3 mo CF rats than moribund CF rats (Fig. S2F), while *Escherichia-Shigella* and *Turicibacter* were more abundant in <3 mo CF rats than moribund rats (Fig. S2G). Despite this shift, traditional CF pathogens such as *P. aeruginosa* and *S. aureus* remained, with an increased relative abundance of *Staphylococcaceae*, *Streptococcaceae*, *and Veillonellaceae* observed in the <3 mo CF rats and an increased relative abundance of *Xanthomonadaceae and Pseudomonadaceae* observed in the >3 mo CF rats (Fig. S3). Along with the shift in bacterial community composition, a non-significant, trending increase in microbial diversity was observed in the <3 mo and >3 mo CF rats as compared to moribund rats (Fig. 4D). Data from microbiome analysis confirmed the results we found from the identification of bacteria from lung homogenate through 16S rRNA PCR (Table 5). Taken together, these data indicate that post-DIOS, the lung microbiome exhibits an increase of fecal microbial communities, but key CF pathogens are still present with age-based differences that recapitulate the evolution of the colonization landscape in pwCF (7).

**TABLE 5:** Bacteria cultured from lung homogenate of WT and CF rats^*a*^

Group	Bacteria cultured from lung	Primary bacterial classification
WT (*n* = 5)	*Delftia tsuruhatensis*	Environmental
	*Delftia lacustris*	Environmental
	*Acinetobacter oryzae*	Environmental
	*Pseudomonas putida*	Environmental
	*Pseudomonas geniculata*	Environmental
	*Staphylococcus epidermidis*	Commensal
	*Staphylococcus hominis*	Commensal
	*Stenotrophomonas* sp.	Traditional CF pathogen
<3 mo (*n* = 11)	*Mammaliicoccus sciuri*	Commensal
	*Klebsiella oxytoca*	Fecal
	*Escherichia coli*	Fecal
	*Enterococcus faecalis*	Fecal
	*Proteus mirabilis*	Fecal
	*Staphylococcus aureus*	Traditional CF pathogen
	*Pseudomonas aeruginosa*	Traditional CF pathogen
	*Stenotrophomonas maltophilia*	Traditional CF pathogen
>3 mo (*n* = 11)	*Priestia aryabhattai*	Environmental
	*Paenibacillus* sp.	Environmental
	*Staphylococcus hominis*	Commensal
	*Staphylococcus epidermidis*	Commensal
	*Micrococcus* sp.	Commensal
	*Staphylococcus nepalensis*	Commensal
	*Micrococcus luteus*	Commensal
	*Mammaliicoccus sciuri*	Commensal
	*Staphylococcus lentus*	Commensal
	*Enterococcus* sp.	Fecal
	*Klebsiella* sp.	Fecal
	*Enterococcus faecalis*	Fecal
	*Escherichia coli/fergusonii*	Fecal
	*Proteus mirabilis*	Fecal
	*Pseudomonas aeruginosa*	Traditional CF pathogen

^*a*^ Lung homogenate from WT, <3 mo CF, and >3 mo CF rats grew environmental microbes, commensal pathobionts, fecal microbes, and traditional CF pathogens.

### The relative abundance of *P. aeruginosa* is greater in >3 mo CF rats than WT rats following induced chronic infection

**Fig 5:**
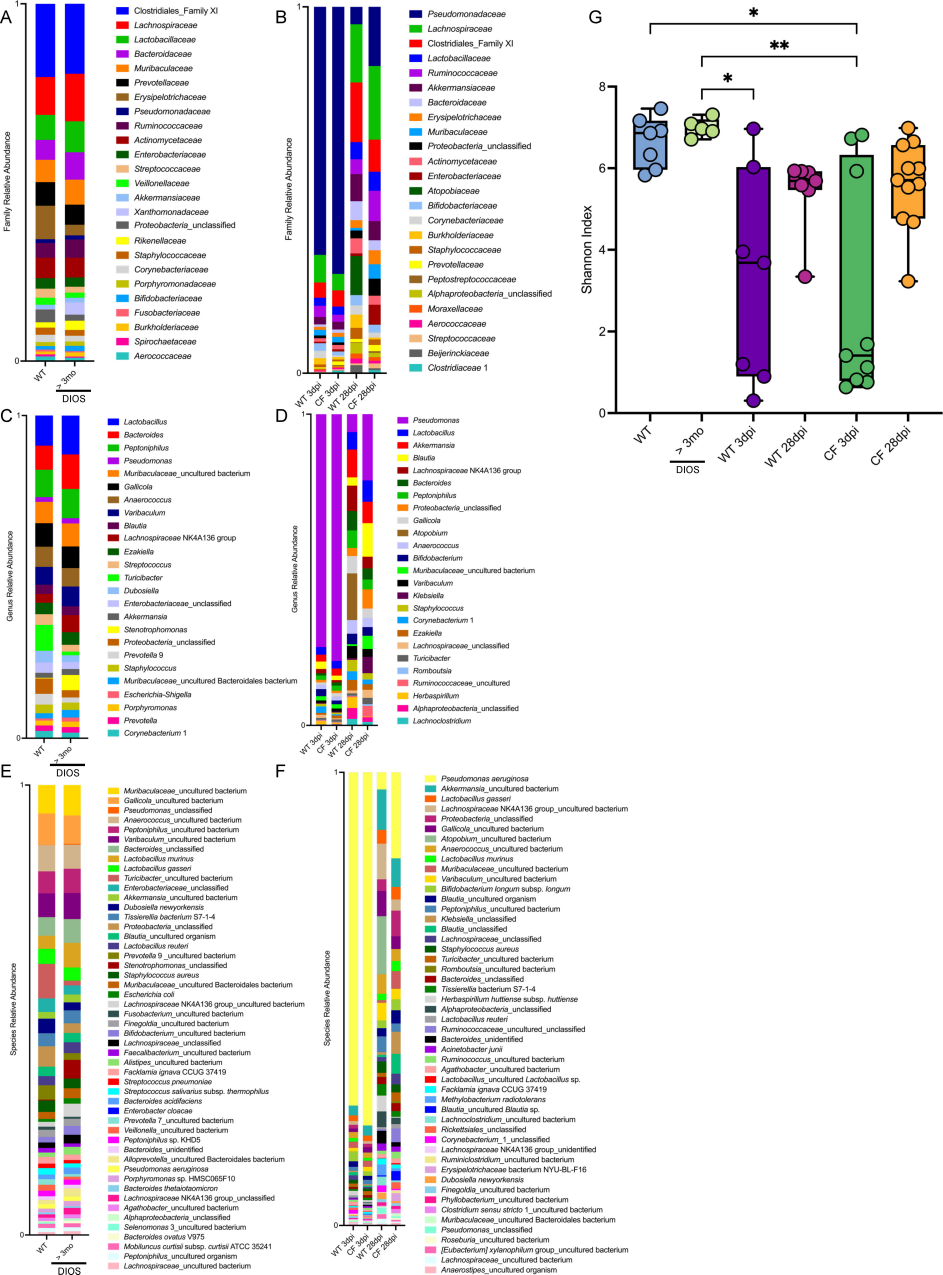
>3 mo CF rats have a higher relative abundance of *P. aeruginosa* in the lung as compared to WT rats following induced chronic infection. Relative family (**A and B**) and genus (**C and D**) level taxonomic abundance as determined by ASVs are shown. The top 25 ASVs excluding mitochondria are included. Relative species (**E and F**) level taxonomic abundance as determined by ASVs is shown. The top 50 ASVs excluding mitochondria are included. Alpha diversity (**G**) measured by the Shannon index is reported. Data are shown as maximum, median, minimum, and IQR and analyzed via Kruskal-Wallis test with Dunn’s multiple comparison test. *P < 0.05, **P < 0.01. WT *n* = 7, >3 mo *n* = 5, WT 3 dpi *n* = 7, CF 3 dpi *n* = 9, WT 28 dpi *n* = 7, CF 28 dpi *n* = 11.

Our lab has previously shown that CF rats at least 6 months of age remain infected with *P. aeruginosa* for up to 28 days post-infection (dpi), while their WT agemates clear *P. aeruginosa* by 7 dpi (25). We aimed to understand how induced *P. aeruginosa* infection impacts the lung microbiome. Both *Pseudomonas_*unclassified and *P. aeruginosa* were present in healthy WT rats and spontaneously colonized >3 mo CF rats (Fig. 5E). Following induced *P. aeruginosa* infection, *P. aeruginosa* composed the highest relative abundance of all bacterial species in both WT and >3 mo CF rats at the 3 dpi acute infection time point (Fig. 5F). By the induced 28 dpi chronic infection time point, the relative abundance of *P. aeruginosa* was increased in the lungs of >3 mo CF rats as compared to WT rats (Fig. 5F). Despite many members of the microbiome being consistent, induced infection with *P. aeruginosa* changed a number of the bacterial family and genus members present compared to healthy WT and spontaneously colonized >3 mo CF rats (Fig. 5A through D). As compared to healthy WT, microbial diversity was reduced at the 3 dpi acute infection timepoint in both WT and CF rats but unchanged at 28 dpi (Fig. 5G). These data confirm the previous findings of our lab concerning induced *P. aeruginosa* infection. *P. aeruginosa* is similar in relative abundance at 3 dpi between the WT and >3 mo CF rats; however, by 28 dpi, *P. aeruginosa* is increased in relative abundance in >3 mo CF rats as compared to WT rats. Additionally, induced infection with *P. aeruginosa* changes the lung microbiome, with decreasing lung microbial diversity following acute infection.

### Ivacaftor therapy changes the lung and fecal microbiomes

**Fig 6:**
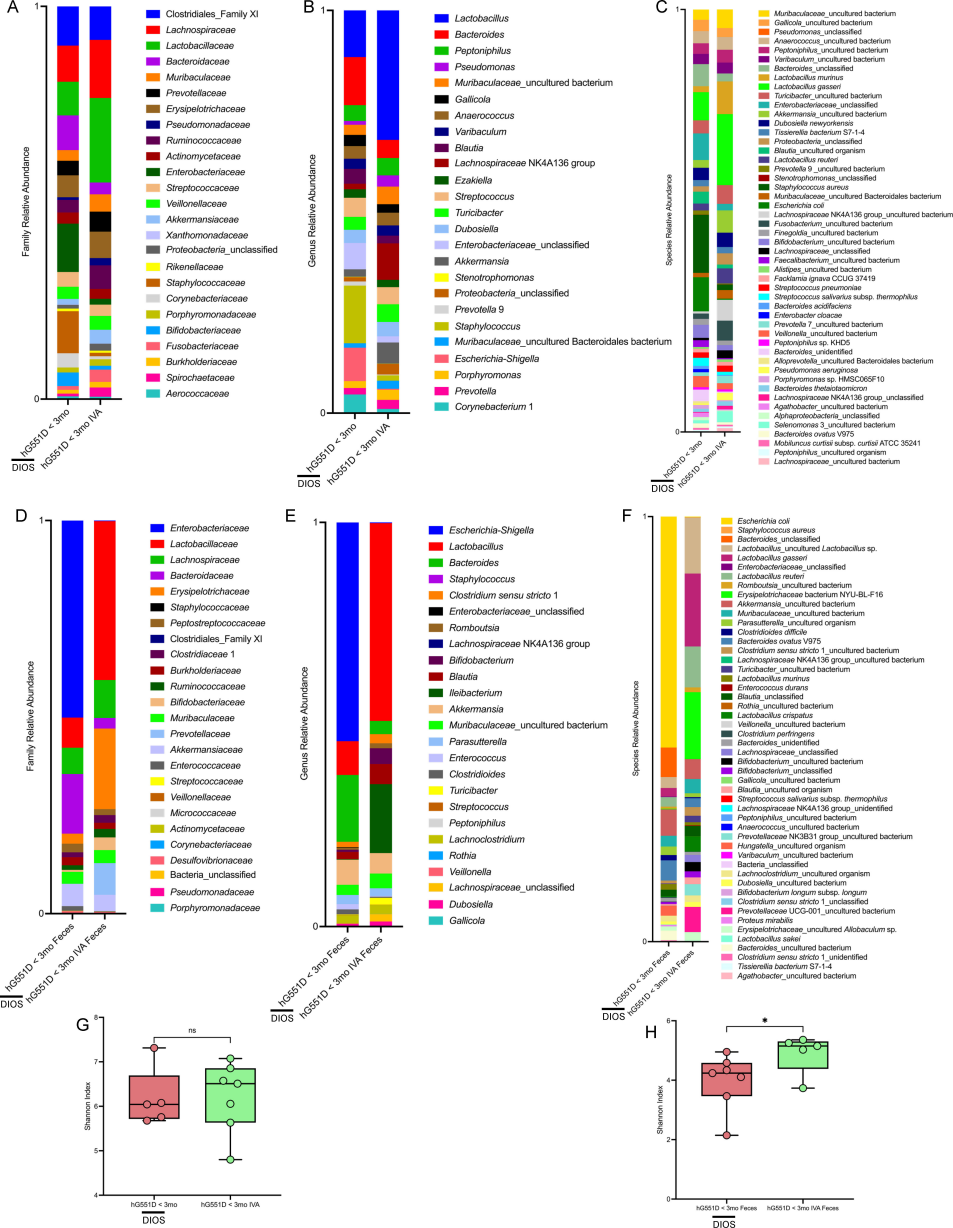
Ivacaftor treatment induces changes to the lung and fecal microbiome of the hG551D CF rat. Relative family level taxonomic abundance of lung tissue (**A**) and feces (**D**) as determined by ASVs is shown. Relative genus level taxonomic abundance of lung tissue (**B**) and feces (**E**) as determined by ASVs is shown. The top 25 ASVs excluding mitochondria and chloroplast are included. Relative species level taxonomic abundance of lung tissue (**C**) and feces (**F**) as determined by ASVs is shown. The top 50 ASVs excluding mitochondria and chloroplast are included. Alpha diversity of lung tissue (**G**) measured by the Shannon index is reported. Data are shown as maximum, median, minimum, and IQR. Data are ns, determined by unpaired *t* test. Alpha diversity of feces (**H**) measured by the Shannon index is reported. Data are shown as maximum, median, minimum, and IQR. *P < 0.05. Lung hG551D < 3 mo *n* = 5, lung hG551D < 3 mo IVA *n* = 7, feces hG551D < 3 mo *n* = 7, feces hG551D < 3 mo IVA *n* = 5.

A number of the <3 mo CF rats with DIOS were of the genotype humanized-G551D. Similar to pwCF, this mutation in our rat model has been shown to be responsive to ivacaftor therapy (13, 22). We aimed to investigate the impact of ivacaftor treatment on the microbiome of the lung and feces. We administered ivacaftor to a subset of moribund hG551D rats and found that it induced changes to the lung microbiome at the family (Fig. 6A), genus (Fig. 6B), and species (Fig. 6C) taxonomic levels. Notably, the relative abundance of *Staphylococcaceae* (Fig. S2H) decreased with ivacaftor treatment, with a corresponding trending decrease in *S. aureus* (Fig. S2I). These changes were not associated with a change in microbial diversity of the lung (Fig. 6G), although it is not clear whether this result corresponds to physiologic changes previously seen in the hG551D lung (13). We saw the most dramatic changes in the fecal microbiome at the family (Fig. 6D), genus (Fig. 6E), and species (Fig. 6F) taxonomic levels, namely a sharp decrease in the relative abundance of *E. coli* after ivacaftor treatment (Fig. 6F). Increased fecal microbial diversity was observed following ivacaftor treatment (Fig. 6H). These data indicate that ivacaftor treatment results in changes to the microbiome of the lung and feces, with the most dramatic changes observed in the fecal microbiome.

## DISCUSSION

**Fig 7:**
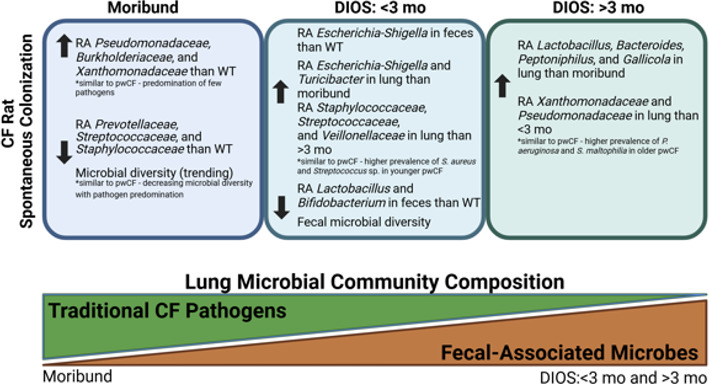
Summary figure. The timing and composition of the lung microbial community are depicted across the disease spectrum of the CF rat.

Many animal models of CF have been developed to study the complex disease pathophysiology. However, every model is not fully replicative of human CF disease. Thus far, only the CF pig and ferret have been shown to develop spontaneous lung infection, a hallmark complication of CF (26, 27). The CF rat has been shown to develop the CF airway mucus defect by 6 months of age, including increased mucus viscosity and decreased MCT rate, corresponding with submucosal gland hypertrophy (21). These mucus defects correspond with the ability of aged CF rats to remain infected for up to 28 dpi (25). Until now, changes to the lung microbiome have not been demonstrated in this model. We show here the ability of the CF rat to become spontaneously colonized with traditional CF-associated bacteria, including *P. aeruginosa*, *S. aureus*, *S. maltophilia*, *Prevotella* sp., and *S. pneumoniae* (Fig. 7).

Studies investigating the link between *CFTR* mutation type and the gut microbiome are more prevalent than the lung microbiome. A study of pwCF represented 26 *CFTR* genotypes, categorized into homozygous-F508del, heterozygous-F508del, and non-F508del, found that both genotype and disease severity were associated with changes to the composition of the fecal microbiome (28). Furthermore, a study examining the gut microbiome of children with CF who were grouped as either pancreatic sufficient or insufficient, based on mutation class, found a small effect of CFTR dysfunction severity on the gut microbiome (29). Although studies of CF genotype on the lung microbiome are limited, Muhlebach et al. found that in sputum samples from pwCF, genotype (number of F508del alleles) was not associated with the presence of anaerobic bacteria, but the number of F508del alleles was positively associated with *Pseudomonas* prevalence (30). Our data show that there is a similar composition of the lung microbiome between the three *CFTR* genotypes represented by our rat model, likely due to the highly controlled experimental environment able to be achieved in the rat experiments.

In healthy individuals, the gut microbiome is dominated by *Bacteroidetes* and *Firmicutes*, while pwCF exhibit a reduced microbial diversity and increased abundance of *E. coli* (31). Our data show that feces from <3 mo CF rats (42.4%) have a 180% difference in the relative abundance of *Enterobacteriaceae* as compared to WT feces (2.2%). Additionally, germ-free CF mice subjected to fecal microbiota transplants from non-CF mice showed a different microbial profile post-transplant than the non-CF controls (32). This suggests that CFTR dysfunction alone impacts dysbiosis of the gut microbiome, a finding that data from our CF rat model also supports. This work did not investigate the impact of the immune system on either the local microbiome or the gut-lung axis, but we did observe that our <3 mo CF rats exhibit more overlap between the relative abundances of each bacteria taxon at the genus level as compared to WT rats. This could be a result of direct transmission of bacteria from the gut to the lungs, but this requires further study. Additionally, we expect that the microbiome may be changed by the presence of the chronic laxative administration, which is required to reduce the incidence of intestinal obstruction. While controlled by treating the WT rats with laxative as well, we were not able to determine the CF intestinal microbiome in the absence of laxative.

In healthy lung and gut microbiomes, the predominant phyla are *Firmicutes* and *Bacteroidetes* (33). Metagenome sputa analysis from pwCF has shown that *Actinobacteria*, *Bacteroidetes*, *Firmicutes*, *Fusobacteria*, and *Proteobacteria* constitute >99% of the CF airway community (34). Thus, there is some overlap in the lung microbiomes of people without CF and pwCF. The relative abundance of *Bacteroidaceae* in >3 mo CF rats is increased compared to WT rats, while the relative abundance of *Fusobacteriaceae* and *Proteobacteria*_unclassified is decreased in >3 mo CF rats compared to WT rats. Members of *Firmicutes*, including *Lactobacillaceae* and *Lachnospiraceae*, are found in higher relative abundance in the lungs of >3 mo CF rats than WT rats. Thus, all core phyla are found in both healthy WT and CF rats as well as humans with and without CF.

Over the lifespan of pwCF, the lung microbiome, microbial diversity, and disease progression are interconnected. Microbial diversity is lost over time, with younger pwCF having more diverse populations of bacteria than older pwCF (24). Age, antibiotic use, and decreasing lung function significantly correlate with decreased microbial diversity (35). This loss of diversity is also attributed to the establishment of infection by dominant CF pathogens. Direct lung sampling from children with mild CF lung disease revealed infections predominated by conventional CF pathogens including *Staphylococcus*, *Pseudomonas*, *Burkholderia*, *Stenotrophomonas*, and *Escherichia-Shigella* (36). Young moribund rats from our study mirrored spontaneous colonization with these predominating pathogens as well as a trending decrease in microbial diversity.

Before DIOS, moribund CF rats have an increased relative abundance of *Pseudomonas* as compared to WT, <3 mo, and >3 mo rats. Lung infection (37), specifically with *P. aeruginosa*, is an associated co-morbidity for DIOS (38). After DIOS, the relative abundances of traditional CF pathogens in CF rats decreased, coupled with a trending increase in microbial diversity, as the lung microbiome shifted to be predominated by fecal microbes. Nonetheless, key CF pathogens remained in both <3 mo and >3 mo CF rats, with age-based differences in relative abundances similar to those in pwCF. In the <3 mo CF rats, the relative abundances of *Staphylococcaceae*, *Streptococcaceae*, and *Veillonellaceae* were higher than those of the >3 mo CF rats. In the >3 mo CF rats, the relative abundances of *Xanthomonadaceae* and *Pseudomonadaceae* were increased compared to the WT and <3 mo CF rats. These results are similar to what is seen in pwCF, as multi-center studies have shown that nontraditional taxa, such as *Streptococcus* and *Prevotella*, constitute ~50% of the microbiota of those younger than 2 years, whereas in pwCF older than 6 years, traditional CF taxa, such as *Pseudomonas*, *Staphylococcus*, and *Stenotrophomonas* (member of family *Xanthomonadaceae*), predominate (39). Furthermore, our data correspond with what is seen clinically in pwCF, with *S. aureus* being more prevalent in early life and *P. aeruginosa* increasing prevalence in late life (7).

Our lab has recently demonstrated the ability of older CF rats to remain infected with *P. aeruginosa* for up to 28 dpi following induced infection, while WT agemates clear infection by 7 dpi (25). The data shown here strengthen this finding by demonstrating a similar relative abundance of *P. aeruginosa* at 3 dpi between WT and CF rats and an increased relative abundance of *P. aeruginosa* in CF rats at the 28 dpi time point. Additionally, we observed a decrease in microbial diversity at the 3 dpi time point following induced *P. aeruginosa* infection, likely due to *P. aeruginosa* being the predominant microbe in the lung. Microbial diversity continued to trend lower than baseline WT and >3 mo CF rats at the 28 dpi time point, though this difference was not statistically significant. Rats with induced *P. aeruginosa* infection were colonized by members of families such as *Atopobiaceae*, *Peptostreptococcaceae*, and *Moraxellaceae*, which were not present in the top 25 taxa at baseline. Members of these families are described to be found in the microbiome of pwCF (23, 40, 41). Furthermore, induced infection with *P. aeruginosa* led to the loss of *Porphyromonas* and *Stenotrophomonas* from the top 25 taxa, while *Klebsiella*, a rare pathogen among pwCF (42), emerged following induced *P. aeruginosa* infection. In pwCF, chronic infection with *P. aeruginosa* is associated with lung microbiome dysbiosis (43). Induced *P. aeruginosa* infection of our CF rat model similarly led to dysbiosis of the lung microbiome from baseline.

Studies examining the lung microbiome and ivacaftor treatment in pwCF show largely consistent results. The GOAL (G551D Observation-AL) study found that *P. aeruginosa* sputum culture positivity was reduced in the year following initiation of ivacaftor (44). Additionally, while sputum bacterial diversity and total bacterial load did not change after ivacaftor, combined relative abundance of CF pathogens trended towards a nonsignificant decline, *Prevotella* relative abundance increased, and the percent of *P. aeruginosa* positive sputum samples decreased (17). Likewise, Hisert et al. found that ivacaftor treatment reduced sputum *P. aeruginosa* density for up to a year following ivacaftor induction. However, *P. aeruginosa* density rebounded, and no participant eradicated the bacteria (45). Interestingly, ivacaftor treatment in our hG551D <3 mo rats led to an increase in the relative abundance of *Pseudomonadaceae* in the lungs as compared to untreated hG551D <3 mo rats. However, the relative abundance of *Staphylococcus* decreased after ivacaftor administration. There was no change in microbial diversity after ivacaftor treatment, which is consistent with human studies (46). Regarding the gut microbiome, ivacaftor treatment in pwCF was associated with an increased abundance of *Akkermansia* and a decreased abundance of *Enterobacteriaceae*, favoring a healthier gut microbiome (20). We also found a decreased relative abundance of *Enterobacteriaceae*, specifically *E. coli*, in the feces of ivacaftor-treated <3 mo hG551D rats. We found increased relative abundances of *Lactobacillus*_uncultured *Lactobacillus* sp., *Lactobacillus gasseri*, and *Lactobacillus reuteri* in feces of the ivacaftor-treated <3 mo hG551D rats. While we did not observe an increase in the relative abundance of *Akkermansia* in feces of ivacaftor-treated <3 mo hG551D rats, there was an increase in the relative abundance in the lung. We also observed an increase in the microbial diversity of the feces upon ivacaftor administration. Taken together, these data indicate that ivacaftor treatment in the hG551D rat is associated with beneficial changes to the microbial communities in the gut and lung, including increased microbial diversity of the feces.

In summary, we present the first evidence for spontaneous colonization in the CF rat model with traditional CF-associated pathogens. We provide evidence for spontaneous lung colonization preceding the development of DIOS in this model before a shift to a lung microbiome predominated by fecal microbes. We show that induced *P. aeruginosa* infection leads to lung microbiome dysbiosis in the CF rat. Additionally, we demonstrate that ivacaftor treatment results in changes to the lung microbiome, and to a greater extent, the fecal microbiome. Further studies are needed to fully explore the gut-lung axis in this model, thereby elucidating the exact contribution of lung colonization to DIOS development and the subsequent shift to fecal microbes in the lung. Efforts are currently underway to pinpoint CFTR dysfunction to only one organ using conditionally expressed models that could identify the origins of the microbiome in each.

## MATERIALS AND METHODS

### CF rat model

All animal experiments at the University of Alabama at Birmingham (UAB) were conducted in accordance with UAB IACUC-approved protocols. All experiments used Sprague-Dawley CFTR Δ16 bp mutation (termed KO), G542X CFTR, or humanized-G551D CFTR rats or their littermate WT controls, CFTR^+/+^ (termed WT). These rat strains were bred and genotyped as described previously (11–13). Litters remained with lactating dams until weaning at 21 days post-birth. Animals were bred and housed in standard cages with a 12-h light/dark cycle in temperatures between 71°F and 75°F. Animals maintained *ad libitum* access to food and water. WT and CF rats of the same sex were cohoused from the time of weaning to study conclusion. Post-weaning, rats were maintained on a standard rodent diet supplemented with DietGel 76A (Clear H_2_O, Westbrook, ME, USA) and water containing 50% Go-LYTELY (Braintree Laboratories, Inc., Braintree, MA, USA) to reduce mortality from GI obstruction. Animals used in this study were classified as either <3 mo of age or >3 mo of age to correspond with mucus defect phenotype.

### Identification of moribund or DIOS-affected rats

CF rats with DIOS (<3 mo and >3 mo) were identified by lethargy, excess porphyrin discharge around eyes and nose, unkempt coat, rapid weight loss, distended abdomen from intestinal obstruction, and diarrhea. Moribund CF rats were identified as declining rats with the symptoms listed above except obstruction and, therefore, likely to develop DIOS in the future. All WT rats were healthy.

### Ivacaftor administration

A subset of moribund <3 mo hG551D rats received administration of ivacaftor, obtained from Selleckchem (Houston, TX, USA), and suspended in 3% methylcellulose, upon onset of symptoms. Rats were treated for 7 days with 40 mg/kg/day by oral gavage.

### Lung collection

Rats were euthanized via intraperitoneal injection of 500 µL pentobarbital sodium (390 mg/mL). Rats were exsanguinated by severance of the hepatic portal vein. The thoracic cavity was exposed, and lungs were removed. Left lungs were mechanically homogenized in 5 mL of Ham’s Nutrient Mix F-12 media (Gibco, Thermo Fisher Scientific, Waltham, MA, USA) and plated for bacterial identification. Right lungs were stored at −80°C until microbiome analysis.

### Feces collection

Rats were euthanized as above. The abdominal cavity was exposed, and feces from the ileal-cecal junction were scooped into a sterile tube to minimize contamination.

### Culture of unknown lung bacteria

About 100 µL of left lung homogenate was spread onto Luria-Bertani (LB) agar, Miller (Fisher BioReagents Microbiology Media, Fisher Scientific, Waltham, MA, USA). Plates were incubated at 37°C for 72 h. Individual colonies that appeared phenotypically different were isolated on separate LB plates and incubated at 37°C for 24 h.

### DNA extraction of unknown bacteria

Individual colonies of each bacterium from lung homogenate were inoculated into 5 mL of tryptic soy broth (TSB) and grown overnight in an incubator at 37°C with continuous shaking. Bacteria were harvested through centrifugation at 5,000 rpm for 10 min. The supernatant was discarded, and pellets were resuspended in 80 µL of lysis buffer containing Tris-HCL (Thermo Scientific Chemicals, Fisher Scientific, Waltham, MA, USA), EDTA (Invitrogen, Thermo Fisher Scientific, Waltham, MA, USA), and Triton X-100 (Sigma-Aldrich, Millipore Sigma, St. Louis, MO, USA). Pellets were then treated with 20 µL of 25 mg/mL lysozyme in Mili-Q H_2_O (Thermo Fisher Scientific, Waltham, MA, USA). DNA was extracted from bacterial cell lysate following the protocol of the Monarch Genomic DNA Purification Kit (New England Biolabs, Inc., Fisher Scientific, Waltham, MA, USA). DNA was eluted with UltraPure DNase/RNase-Free Distilled Water (Invitrogen, Thermo Fisher Scientific, Waltham, MA, USA).

### Identification of unknown bacteria

DNA was subjected to polymerase chain reaction (PCR) to amplify the 16S rRNA gene for species identification of bacterial isolates. DNA was added to a mixture containing Promega GoTaq DNA Polymerase (Promega M3001, Fisher Scientific, Waltham, MA, USA), 5× colorless GoTaq Reaction Buffer (Promega M3001, Fisher Scientific, Waltham, MA, USA), dNTP Mix (10 mM each) (Thermo Fisher Scientific, Waltham, MA, USA), and UltraPure DNase/RNase-Free Distilled Water (Invitrogen, Thermo Fisher Scientific, Waltham, MA, USA). Forward and reverse primer sequences were utilized from previously published work on 16S rRNA sequencing, forward primer 63f (5′-CAG GCC TAA CAC ATG CAA GTC-3′) and reverse primer 1387r (5′-GGG CGGWGTGTACAA GGC-3′) (47). PCR products were sent to The Genomics Core Laboratory of the Heflin Center for Genomic Science at UAB for Sanger Sequencing. Sequencing results were processed with the NIH NCBI Nucleotide Basic Local Alignment Search Tool (BLAST) to identify bacterial species.

### Culture of *P. aeruginosa*

Rats modeling induced infection were infected with mucoid *P. aeruginosa* strain PAM57-15, a clinical isolate used previously (25, 48, 49). Bacteria were grown in TSB (Millipore Sigma, St. Louis, MO, USA) overnight in an incubator at 37°C with continuous shaking. Once bacteria reached A600 of 1.0–1.5, they were harvested through centrifugation and resuspended in 6.5 mL of phosphate-buffered saline (PBS). Bacteria were embedded in agarose using previously established methods and adjusted to a concentration of approximately 3 × 10^6^ colony-forming units (CFUs)/300 µL (25).

### Induced infection with *P. aeruginosa* agarose beads

*P. aeruginosa*-laden agarose beads were administered via intratracheal inoculation at a concentration of approximately 3 × 10^6^ CFUs/300 µL. Rats were anesthetized by isoflurane gas and suspended by their incisors on an intubation stand (Braintree Scientific, Inc., Braintree, MA, USA). The tongue was pulled out and forward using blunt forceps. A 1 mL syringe attached to a blunt 18-gauge luer stub (Instech Laboratories, Plymouth Meeting, PA, USA) was inserted past the tongue and into the trachea to ensure delivery into the lungs. The inoculum was followed by 50 µL of air. Rats remained upright on the intubation stand for at least 20 s to complete inhalation of the inoculum and were then removed to their cage to recover. Rats were euthanized via pentobarbital injection at days 3 and 28 post-infection, and lungs were collected as described above.

### Microbiome analysis

Frozen lung tissue was sectioned into 100 mg pieces. Tissue was dissected and covered with 100 µL of PBS before delivery to the core for processing. Feces underwent no preprocessing prior to delivery to the core. Microbiome analysis was performed by the UAB Microbiome Institutional Research Core as previously described (50). Briefly, DNA was extracted, and PCR was used for amplification of the V4 region of the 16S rRNA gene to create an amplicon library. The PCR products were sequenced using the NextGen sequencing Illumina MiSeq platform. The microbiome analysis package QWRAP (QIIME [Quantitative Insights Into Microbial Ecology] wrapper) was used for analysis.

## Data Availability

The data from this study have been deposited in the NIH Sequence Read Archive (SRA) as BioProject PRJNA1221303.

## SUPPLEMENTAL MATERIAL

Supplemental material mbio.03883-24-s0001.pdf Figures S1 to S3.

ASM does not own the copyrights to Supplemental Material that may be linked to, or accessed through, an article. The authors have granted ASM a non-exclusive, world-wide license to publish the Supplemental Material files. Please contact the corresponding author directly for reuse.
