# Supplementary material for: Spontaneous lung colonization in the cystic fibrosis rat model is linked to gastrointestinal obstruction
Source: mBio. 2025 Mar 5;16(4):e03883-24. doi: 10.1128/mbio.03883-24 (PMC11980572; doi:10.1128/mbio.03883-24)
Supplement: Supplemental material — Figures S1 to S3. [file mbio.03883-24-s0001.pdf]

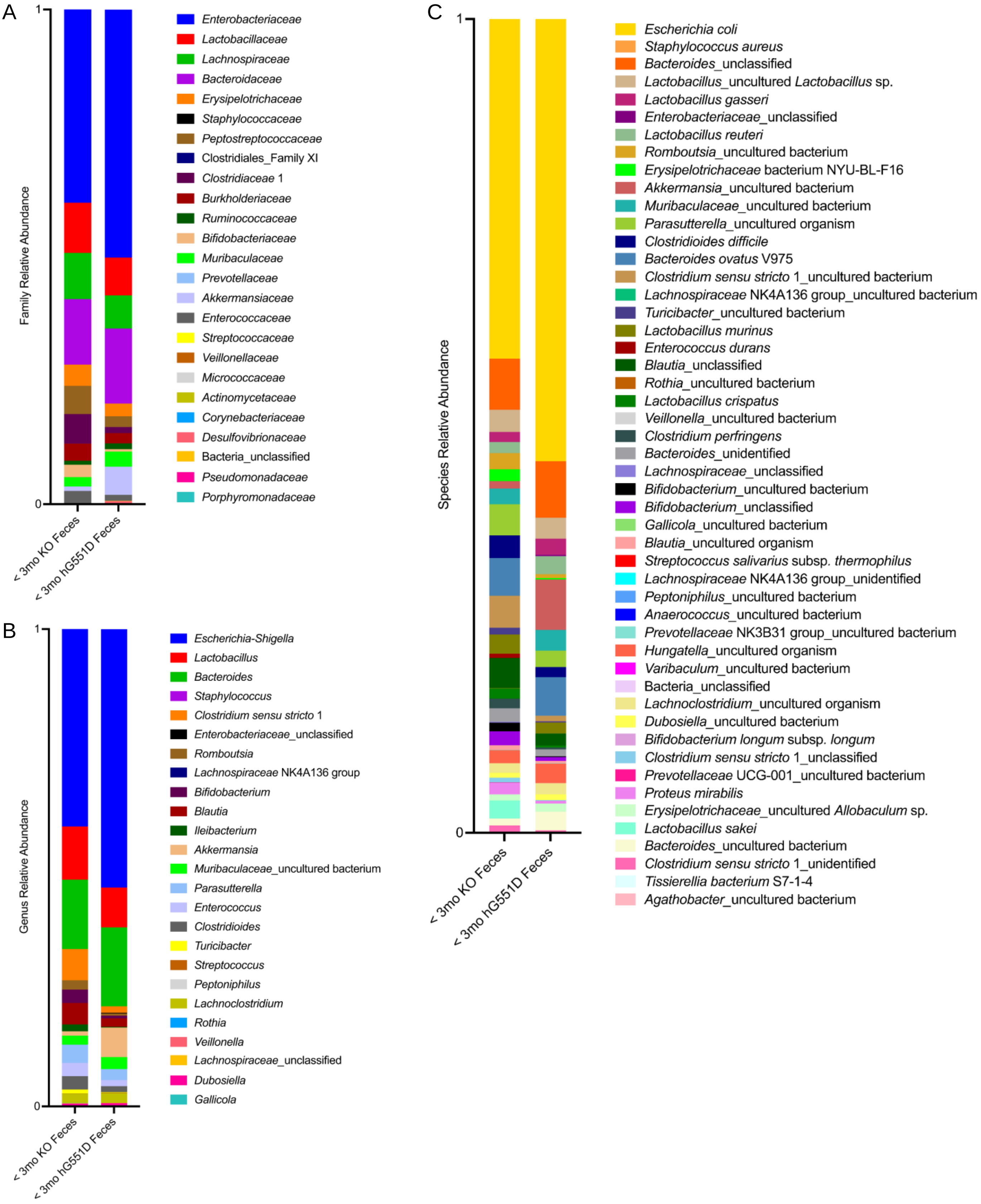

Figure S1: Fecal microbiome is not dependent on CFTR genotype. Relative family (A) and genus (B) level taxonomic abundance as determined by amplicon sequence variants (ASVs) are shown. The top 25 ASVs excluding mitochondria are included. Relative species (C) level taxonomic abundance as determined by ASVs is shown. The top 50 ASVs excluding mitochondria are included. <3 mo KO Feces n = 7, <3 mo hG551D Feces n = 3.

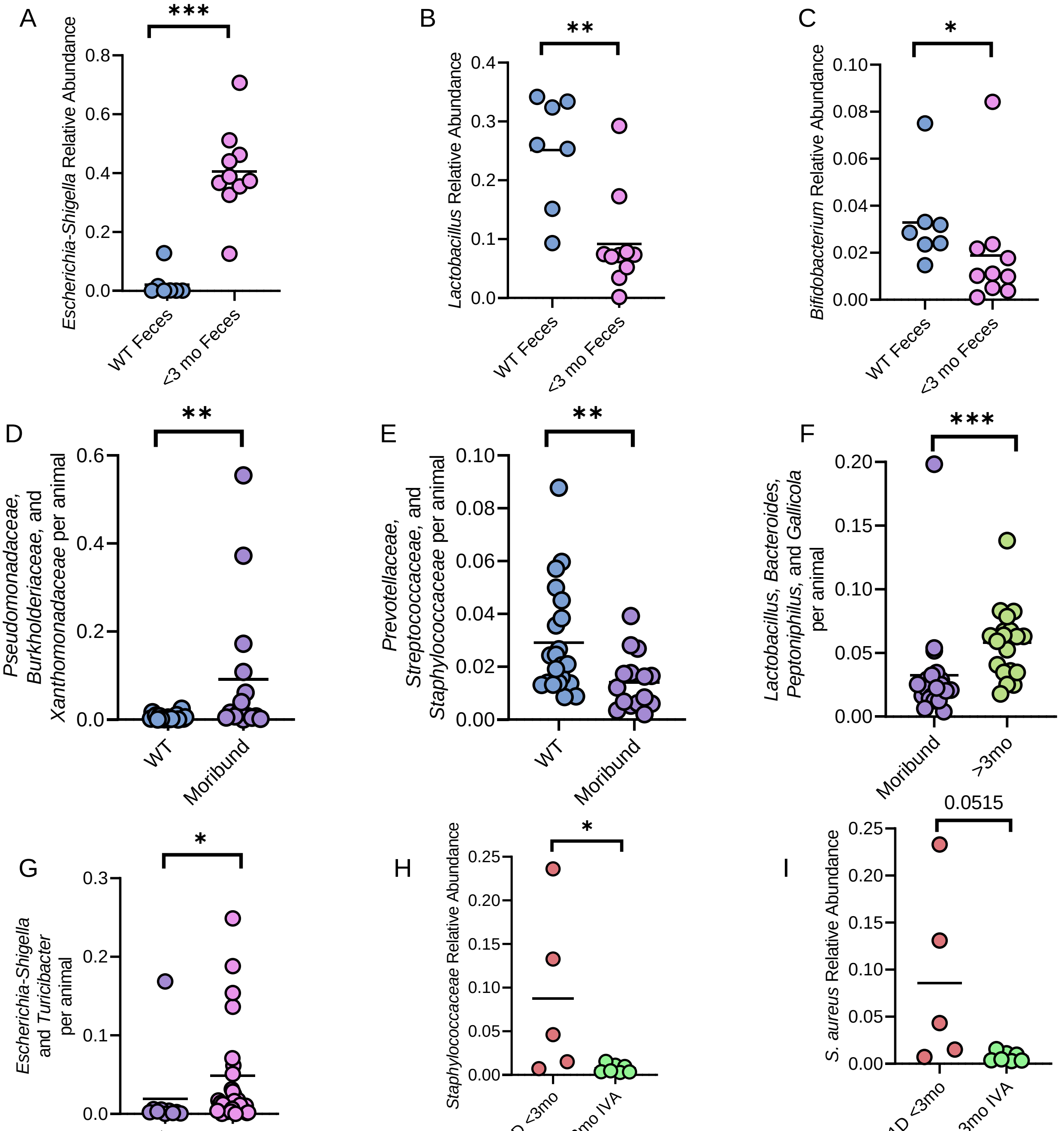

Figure S2: Changes in the relative abundance of key microbial taxa were observed in the lungs and feces. Fecal relative abundance of (A) *Escherichia-Shigella*, (B) *Lactobacillus*, and (C) *Bifidobacterium* differed between WT and <3 mo CF rats. Relative abundance of traditional CF pathogens in the lung were totaled for WT and Moribund rats. *Pseudomonadaceae, Burkholderiaceae*, and *Xanthomonadaceae* (D) composed a greater proportion of the total relative abundance of Moribund rats as compared to WT rats, while *Prevotellaceae*, *Streptococcaceae*, and *Staphylococcaceae* (E) composed a lower proportion. There was an increased relative abundance of fecal-associated microbes in the lungs of >3 mo and <3 mo CF rats than moribund CF rats. *Lactobacillus, Bacteroides, Peptoniphilus*, and *Gallicola* (F) exhibited increased relative abundance in >3 mo CF rats, while *Escherichia-Shigella* and *Turicibacter* (G) had an increased relative abundance in <3 mo CF rats. Data analyzed via Mann-Whitney test. Ivacaftor treatment in hG551D rats impacted the relative abundance of (H) *Staphylococcaceae* and (I) *S. aureus.* Data analyzed via unpaired t test. *P<0.05, **P<0.01, ***P<0.001. WT Feces n = 7, <3 mo Feces n = 10, WT n = 7/taxa, Moribund n = 5/taxa, >3 mo n = 5/taxa, <3 mo n = 20/taxa. hG551D <3 mo n = 5, hG551D <3 mo IVA n = 7.

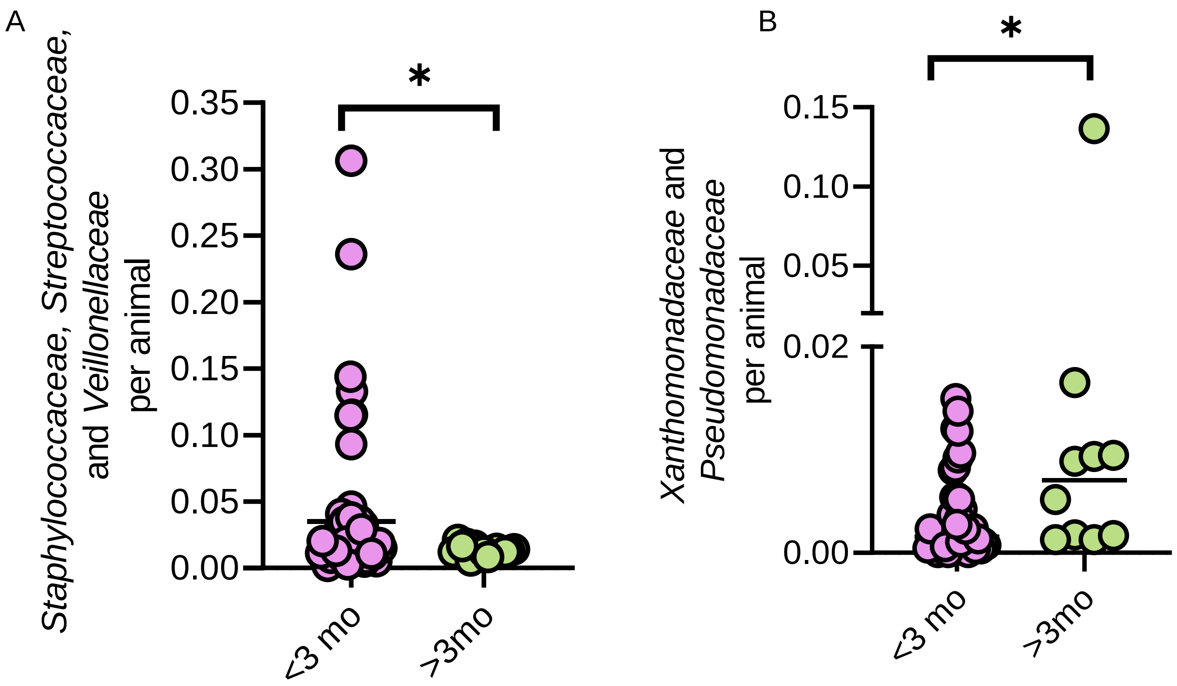

Figure S3: Age-based differences in traditional CF pathogens in the lung exist post-DIOS. Lung relative abundance of traditional CF pathogens (A) *Staphylococcaceae, Streptococcaceae,* and *Veillonellaceae* and (B) *Xanthomonadaceae* and *Pseudomonadaceae* differ between <3 mo and >3 mo CF rats after DIOS. Data analyzed via Mann-Whitney test. *P<0.05. >3 mo n = 5/taxa, <3 mo n = 20/taxa.
